# Modulation of Hippocampal Antioxidant Defense System in Chronically Stressed Rats by Lithium

**DOI:** 10.1155/2019/8745376

**Published:** 2019-02-17

**Authors:** Nataša Popović, Vesna Stojiljković, Snežana Pejić, Ana Todorović, Ivan Pavlović, Ljubica Gavrilović, Snežana B. Pajović

**Affiliations:** Institute of Nuclear Sciences “Vinča”, Laboratory of Molecular Biology and Endocrinology, University of Belgrade, Belgrade, Serbia

## Abstract

This study examined the effects of lithium on gene expression and activity of the antioxidant enzymes copper zinc superoxide dismutase (SOD1), manganese superoxide dismutase (SOD2), catalase (CAT), glutathione peroxidase (GPx), and glutathione reductase (GR) in the hippocampus of chronically stressed rats. In addition, we examined the effects of lithium on anxiety behaviors, hippocampal concentrations of dopamine (DA) and malondialdehyde (MDA), protein levels of brain-derived neurotrophic factor (BDNF), tyrosine hydroxylase (TH), dopamine transporter (DAT), and catechol-O-methyltransferase (COMT), as well as activity of monoamine oxidase (MAO) in chronically stressed rats. The investigated parameters were quantified by real-time RT-PCR, Western blot analyses, and assays of enzyme activities. We found that lithium did not change gene expression of SOD1, CAT, GPx, and GR but decreased gene expression of SOD2 in chronically stressed rats. A very important result in this study was that lithium treatment decreased the enzyme activities of SOD1 and SOD2 but increased the enzyme activities of GPx and GR in stress condition, which indicates the control of redox balance. The reduced concentration of MDA confirms this. In addition, we found that lithium treatment decreased high protein levels of BDNF and DAT in chronically stressed rats to the level found in unstressed animals. Also, lithium treatment increased the expression of TH but decreased the enzyme activity of MAO B, which contributed to the increase of hippocampal concentration of DA in chronically stressed rats to the level of unstressed animals. Finally, lithium treatment in animals exposed to chronic stress increased the time spent in open arms. Lithium-induced modulation of hippocampal antioxidant status and attenuation of oxidative stress stabilized behavior in animals with high anxiety index. In addition, reduced oxidative stress was followed by the changes of both turnover of DA and levels of BDNF protein in chronically stressed rats treated with lithium. These findings may be important in preclinical research of the effects of lithium on oxidative stress level in pathological conditions.

## 1. Introduction

Molecular interactions in the neuroendocrine system under stress condition can lead to homeostatic disorders [1, 2]. Chronic stress induces overactivation and dysfunction of stress-activated systems, resulting in further brain damage and mood disorders [3, 4]. One of the key mechanisms for the modulation of brain functions in stress conditions is monoaminergic signaling. In addition, it is known that brain-derived neurotrophic factor (BDNF) modulates the activity of monoaminergic systems in the rat brain [5]. Normal monoaminergic turnover results from balance among synthesis, degradation, release, and reuptake of monoamines. In our previous studies, we found that chronic restraint stress (CRS) induced significant decrease of both hippocampal dopamine (DA) concentration [6] and protein levels of tyrosine hydroxylase (TH), a “rate-limiting” enzyme of dopamine biosynthesis [7], which confirmed that the hippocampus was particularly sensitive to chronic stress [8, 9]. Data about the dynamics of DA transmission and degradation are very important for understanding dopaminergic turnover. The dynamics of DA transmission is regulated by reuptake through dopamine transporter (DAT). Monoamine oxidase (MAO) and catechol-O-methyltransferase (COMT) are enzymes which catalyze the oxidative deamination of monoamine neurotransmitters including DA. The byproducts of these reactions include a number of potentially neurotoxic species, such as hydrogen peroxide and ammonia. Hydrogen peroxide can trigger the production of reactive oxygen species (ROS) and induce mitochondrial damage and neuronal apoptosis. It is known that the brain is particularly vulnerable to oxidative damage since it contains large amounts of polyunsaturated fatty acids and possesses low antioxidant capacity [10, 11]. Malondialdehyde (MDA) is the frequently used biomarker of oxidative stress in many health problems including mood disorders. The literature data have shown that there is a direct involvement of oxidative stress in anxiety-like behavior in rodents [12]. Our earlier research confirmed that chronic restraint stress (CRS) influenced anxiety-like behavior in rats [6]. In the pathophysiology of mood disorders, lithium is known as an effective drug in the long-term stabilization of moods. Also, lithium has a neurotrophic and neuroprotective function and improves total antioxidant activity [13–16]. In our earlier studies, we found that CRS induced increased activity of superoxide dismutase 1 (SOD1), superoxide dismutase 2 (SOD2), and catalase (CAT) in the hippocampus [17]. The increased activity of antioxidant enzymes may be an important adaptive phenomenon of the antioxidant defense system in chronically stressed rats [17]. It is known that treatment with antidepressants significantly decreased the activities of SOD and CAT in depressive patients [18], as well as increased DA levels in the prefrontal cortex [19]. However, very little is known about the antioxidant defense system and turnover of DA in animals with high anxiety index treated with lithium.

Because of the direct involvement of oxidative stress in anxiety-like behavior in stress conditions, detecting the changes of gene expression and activity of the antioxidant enzymes as well as monitoring the changes of dopaminergic turnover in the hippocampus in chronically stressed rats treated with lithium may be very important in the research on the role of lithium in maintaining antioxidant status in pathological conditions. Therefore, in this study we examined gene expression and activity of the antioxidant enzymes SOD, CAT, glutathione peroxidase (GPx), and glutathione reductase (GR), as well as protein levels of BDNF, TH, DAT, and COMT and activity of MAO and concentrations of DA and MDA in the hippocampus of chronically stressed rats treated with lithium. An additional aim of the study was to test anxiety in chronically stressed rats treated with lithium.

## 2. Materials and Methods

### 2.1. Animals and Stress Models

Eleven-week-old Wistar male rats (300-340 g) were maintained under standard laboratory conditions with water and food *ad libitum* and kept three to four per cage [20]. The care was taken to minimize the pain and discomfort of the animals according to the recommendations of the Ethical Committee of the Vinča Institute of Nuclear Sciences, Belgrade, Serbia, which follows the guidelines of the registered “Serbian Society for the Use of Animals in Research and Education.” In accordance with our previous protocol [21], animals were divided into three groups: *CRS group* (*n* = 20) consisted of animals exposed to chronic restraint stress treatment and *CRS+Li group* (*n* = 20) consisted of animals exposed to chronic restraint stress treatment with Li given each day immediately prior to daily restraint. Restraint stress was performed by placing each animal in a 25 × 7 cm plastic bottle as described previously [22]. The animals in these groups were exposed to 2 h of restraint stress every day at random times during the light period of the light/dark cycle to avoid habituation during the experimental procedure of 14 days [23]. Lithium was administered intraperitoneally to the animals, once a day for 14 days as described previously [24]. The initial lithium dose was 1.5 mEq/kg for 2 days and was then increased to 2.3 mEq/kg for 7 days, followed by 3 mEq/kg for 5 days. This lithium administration protocol maintained the plasma lithium concentration above the minimal therapeutic concentration (i.e., 0.4 mM) for the treatment of bipolar disorder throughout the treatment period. Anxiety-like behaviors were assessed by elevated plus maze (EPM) test. Ten animals from each group were tested on the EPM. Animals which were used to test the behavior were not used for further analysis. In order to examine whether lithium decreased high protein levels of BDNF and DAT in chronically stressed rats to the level of unstressed animals, we introduced Control group. The *Control group* (*n* = 10) was not exposed to any treatment. To reduce variance in the physiological parameters due to daily rhythms, the remaining animals (*n* = 10 from each group) were sacrificed at the same time point in the circadian cycle, between 9:00 and 11:00 am, i.e., one day after the last treatments. Animals were sacrificed under no-stress conditions by rapid decapitation. The hippocampuses were rapidly dissected, frozen in liquid nitrogen, and stored at −70°C until analyzed.

### 2.2. Dopamine Measurement

Hippocampus tissues were homogenized in 0.01 N HCl in the presence of EDTA and sodium metabisulfite. Dopamine concentration in hippocampus fractions was determined using 3-CAT Research ELISA kits (Labor Diagnostika Nord, Nordhorn, Germany) according to the manufacturer's protocol. Absorbance was determined at 450 nm using a microplate reader (Stat Fax 2100). Concentrations were normalized to 1 g of tissues in homogenate. Values were expressed as ng of DA per g of *tissues* which is in accordance with our previous protocol [25].

### 2.3. Monoamine Oxidase Enzyme Activities

The determination of MAO B activity was performed using the Amplex Red Monoamine Oxidase Assay (A12214, Molecular Probes, USA), described by Zhou and Panchuk-Voloshina [26]. This assay is based on the detection of H_2_O_2_ in a horseradish peroxidase-coupled reaction using N-acetyl-3, 7-dihydroxyphenoxazine (Amplex Red), a highly sensitive and stable probe for H_2_O_2_. Fluorescence was measured with a fluorometer using excitation at 560 ± 10 nm and fluorescence detection at 590 ± 10 nm. Monoamine oxidase activity was expressed as U/mg of protein as previously described by Gavrilović et al. [25].

### 2.4. RNA Isolation, cDNA Synthesis, and Real-Time RT-PCR

Methods of RNA isolation and cDNA synthesis were described previously by Gavrilović et al. [27]. Total RNAs were isolated from the hippocampal tissue by using TRIZOL reagent (Invitrogen, USA). Reverse transcription was performed using Ready-To-Go You-Prime First-Strand Bead (Amersham Biosciences, UK) and pd (N)_6_ Random Hexamer (Amersham Biosciences, UK) primer according to the manufacturer's protocol, which is in accordance with the protocol of Gavrilović et al. [28]. CuZn SOD (SOD1), Mn SOD (SOD2), CAT, GPx, and GR mRNA levels were quantified by quantitative real-time RT-PCR, as described previously by Gavrilović et al. [27]. TaqMan PCR assays were carried out using Assay-on-Demand Gene Expression Products (Applied Biosystems, USA) for SOD1 (Rn00566938_m1), SOD2 (Rn00690587_g1), CAT (Rn00560930_m1), GPx (Rn00577994_g1), and GR (Rn01482159_m1). The reference gene (endogenous control) was included in each analysis to correct for the differences in the interassay amplification efficiency and all transcripts were normalized to cyclophilin A (Rn00690933_m1) expression [28]. Quantification was done using the 2^−ΔΔCt^ method according to Livak and Schmittgen [29]. The relative expression of the target gene was normalized to cyclophilin A and expressed in relation to the calibrator, i.e., the control sample as previously described by Gavrilović et al. [28].

### 2.5. Hippocampal Tissue Homogenization, Measurement of the Protein Concentration, and Western Blot Analysis

The hippocampus was homogenized in 0.05 M sodium phosphate buffer (pH 6.65). Subsequently, the protein concentration was determined using BCA method (Thermo Scientific Pierce, USA), described by Stich [30]. CuZn SOD (SOD1), Mn SOD (SOD2), CAT, GPx, GR, BDNF, TH, DAT, and COMT proteins were assayed by Western blot analysis as described previously by Gavrilović et al. [27]. Antibodies used for the quantification of specific proteins were as follows: SOD1 (SOD-101, Stressgen, USA), SOD2 (SOD-110, Stressgen, USA), CAT (Calbiochem, Germany), GPx (sc-30147 Santa Cruz Biotechnology, USA), GR (sc-32886, Santa Cruz Biotechnology, USA), BDNF (ab6201, Abcam, USA), TH (ab51191, Abcam, USA), DAT (ab18548, Abcam, USA), and *β*-actin (ab8227, Abcam, USA). After washing, the membranes were incubated in the secondary anti-rabbit (dilution 1 : 5000, Amersham ECL™ Western Blotting Analysis System, UK) antibodies conjugated to horseradish peroxidase. A secondary antibody was then visualized by the Western blotting-enhanced chemiluminescent detection system (ECL, Amersham Biosciences, UK). The membranes were exposed to ECL film (Amersham Biosciences, UK). The result was expressed in arbitrary units normalized in relation to *β*-actin, which is in accordance with our previous protocol [27].

### 2.6. Antioxidant Enzyme Activities

SOD, GPx, and GR activities were determined using assays for enzyme activities, as we previously described [31].

#### 2.6.1. Assay of SOD Activity

Total SOD activity was measured using the Oxis Bioxytech^®^ SOD-525™ Assay (Oxis International Inc., Portland, OR, USA). The method is based on the SOD-mediated increase in the rate of autoxidation of reagent 1 (5,6,6a,11b-tetrahydro-3,9,10-trihydroxybenzo[c] fluorene, R1) in aqueous alkaline solution, yielding a chromophore with maximum absorbance at 525 nm. The kinetic measurement of the change in absorbance at 525 nm was performed. One SOD-525 activity unit was defined as the activity that doubles the autoxidation rate of the control blank. CuZnSOD activity was measured as described above, after pretreating samples with ethanol-chloroform reagent (5/3 vol/vol), which inactivates MnSOD. MnSOD activity was then calculated by subtracting CuZnSOD activity from total SOD activity [17].

#### 2.6.2. Assay of CAT Activity

CAT activity was determined by the method of Beutler [32]. The reaction is based on the rate of H_2_O_2_ degradation by catalase contained in the examined samples. The reaction was performed in an incubation mixture containing 1 M Tris-HCl, 5 mM EDTA, pH 8.0 and monitored spectrophotometrically at 230 nm. One unit of CAT activity was defined as 1 *μ*mol of H_2_O_2_ decomposed per minute under the assay conditions [21].

#### 2.6.3. Assay of GPx Activity

GPx activity was assessed using the Oxis Bioxytech GPx-340 Assay (Oxis International Inc., Portland, OR, USA), based on the principle that oxidized glutathione (GSSG) produced upon reduction of an organic peroxide by GPx is immediately recycled to its reduced form (GSH) with concomitant oxidation of NADPH to NADP+. The oxidation of NADPH was monitored spectrophotometrically as a decrease in absorbance at 340 nm. One GPx-340 unit was defined as 1 *μ*mol of NADH oxidized per minute under the assay conditions [21].

#### 2.6.4. Assay of GR Activity

Activity of GR was measured using the Oxis Bioxytech GR-340 Assay (Oxis International Inc., Portland, OR, USA). The assay is based on the oxidation of NADPH to NADP+ during the reduction of oxidized glutathione (GSSG), catalyzed by a limiting concentration of glutathione reductase. The oxidation of NADPH was monitored spectrophotometrically as a decrease in absorbance at 340 nm. One GR-340 unit was defined as 1 *μ*mol of NADH oxidized per minute under the assay conditions [21].

### 2.7. Malondialdehyde Measurement

Malondialdehyde concentration in the hippocampus fractions was determined using Spectrophotometric Assay for Malondialdehyde Bioxytech^®^ MDA-586 (OXIS Health Products Inc., USA) according to the manufacturer's protocol. The MDA-586 method is based on the reaction of a chromogenic reagent, N-methyl-2-phenylindole, with MDA at 45°C. Malondialdehyde concentration was expressed as *μ*M/mg of protein, which is in accordance with our previous protocol [25].

### 2.8. Elevated Plus Maze (EPM)

The EPM consisted of four elevated (50 cm) contralateral arms (50 cm long and 10 cm wide) with two opposing arms containing 40 cm high opaque walls, which is in accordance with our previous protocol [6]. On the day of EPM testing, rats were transported into the testing room one cage at a time and testing alternated between CRS animals and CRS+Li animals. Each rat was placed in a closed arm, facing the center platform and cage-mates started in the same closed arm, which was counterbalanced across trials. Each rat was given 5 min to explore the EPM and then returned to its home cage. The EPM was cleaned thoroughly using Naturally Living Pet Odor Eliminator between each rat. EPM performance was recorded using an overhead video camera for later quantification. Open and closed arm entries were defined as the front two paws entering the arm, and open arm time began the moment the front two paws entered the open arm and ended upon exit. Rats that displayed thigmotaxis and an aversion to the open arms were considered highly anxious [33]. An added measure of anxiety was calculated for the EPM using the following equation, which unifies all EPM parameters into one unified ratio; anxiety index values range from 0 to 1, with a higher value indicating increased anxiety [34–37].

(1) $$\begin{array}{c} \text{Anxiety index}=1-\left[\frac{\left(\text{open arm time}/5\;\min\right)+\left(\text{open arm entry}/\text{total entry}\right)}{2}\right]. \end{array}$$

### 2.9. Data Analysis

The data are presented as means ± S.E.M. Differences of gene expression (mRNA and protein levels) of SOD1, SOD2, CAT, GPx, GR, BDNF, TH, and DAT; activity of enzymes (MAO B, SOD1, SOD2, CAT, GPx, and GR); and concentration of DA and MDA as well as animal behavior between CRS and CRS+Li animals were analyzed by *t*-test. Statistical significance was accepted at *p* < 0.05.

## 3. Results

### 3.1. Changes of Levels of TH Protein, DA Concentrations, BDNF Protein, DAT Protein, COMT Protein, and MAO B Activity in the Hippocampus

**Figure 1:**
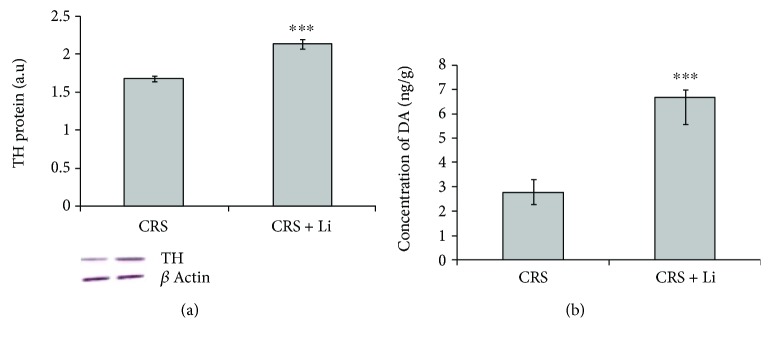
Effects of lithium on tyrosine hydroxylase (TH) protein levels (a) and concentration of dopamine (DA) (b) in the hippocampus of animals exposed to CRS. The values are means ± S.E.M. of 10 rats. Statistical significance: ^∗∗∗^*p* < 0.001 animals exposed to CRS+Li *vs*. CRS animals (*t*-test). The level of TH protein was expressed in arbitrary units normalized in relation to *β*-actin, and the concentration of DA was expressed as ng per gram of tissue (ng/g).

**Figure 2:**
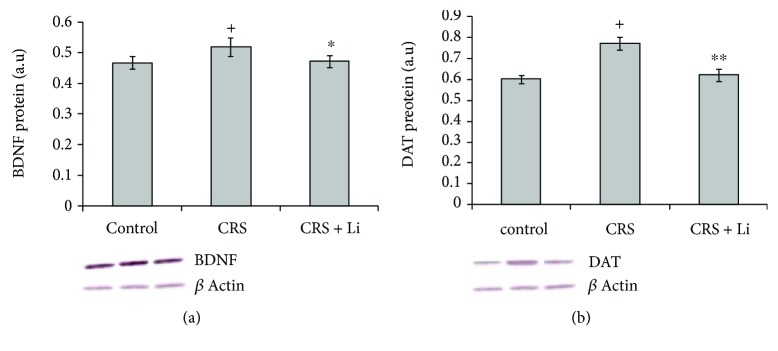
Effects of lithium on brain-derived neurotrophic factor (BDNF) (a) and dopamine transporter (DAT) (b) protein levels in the hippocampus of animals exposed to CRS. The values are means ± S.E.M. of 10 rats. Statistical significance: ^∗^*p* < 0.05, ^∗∗^*p* < 0.01 animals exposed to CRS+Li *vs*. CRS animals (*t*-test); ^+^*p* < 0.05 CRS animals *vs*. Control animals (*t*-test). The result was expressed in arbitrary units normalized in relation to *β*-actin.

**Figure 3:**
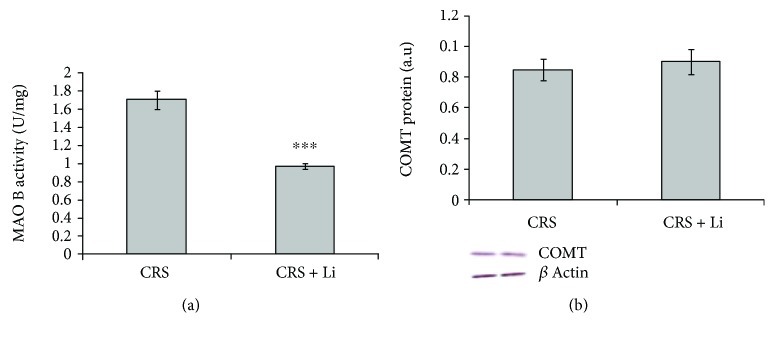
Effects of lithium on monoamine oxidase B (MAO B) enzyme activity (a) and catechol-O-methyltransferase (COMT) protein levels (b) in the hippocampus of animals exposed to CRS. The values are means ± S.E.M. of 10 rats. Statistical significance: ^∗∗∗^*p* < 0.001 animals exposed to CRS+Li *vs*. CRS animals (*t*-test). The level of MAO B activity was expressed as units per milligram of protein (U/mg) and protein levels of COMT were expressed in arbitrary units normalized in relation to *β*-actin.

We found that lithium treatment in animals exposed to CRS significantly increased levels of TH protein by 26% (*p* < 0.001, *t*-test, Figure 1(a)) and increased the concentration of DA by 125% (*p* < 0.001, *t*-test, Figure 1(b)) compared with CRS animals. In addition, lithium treatment decreased levels of BDNF protein by 10% (*p* < 0.05, *t*-test, Figure 2(a)) and decreased levels of DAT protein by 20% (*p* < 0.01, *t*-test, Figure 2(b)) to the level of unstressed animals. Also, the animals exposed to CRS treated with lithium showed decreased levels of MAO B activity by 43% (*p* < 0.001, *t*-test, Figure 3(a)), while levels of COMT protein remained unchanged (Figure 3(b)) compared with CRS animals.

### 3.2. Changes of MDA Concentrations in the Hippocampus

**Figure 4:**
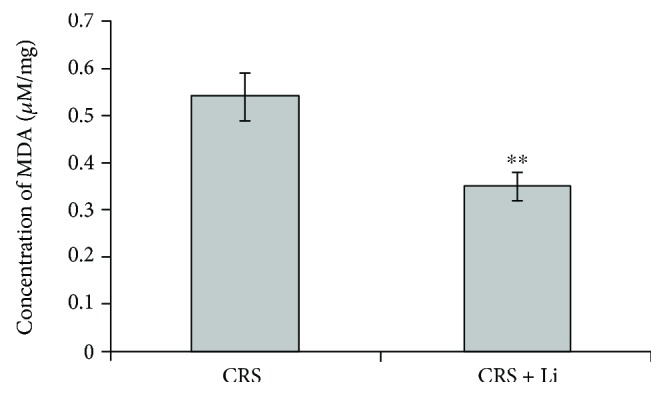
Effects of lithium on the concentration of malondialdehyde (MDA) in the hippocampus of animals exposed to CRS. The values are means ± S.E.M. of 10 rats. Statistical significance: ^∗∗^*p* < 0.01 animals exposed to CRS+Li *vs*. CRS animals (*t*-test). The final result for concentration of MDA was expressed as *μ*M/mg protein.

Lithium treatment decreased MDA concentrations by 35% (*p* < 0.01, *t*-test, Figure 4) compared with CRS animals.

### 3.3. Changes of SOD1 mRNA Levels, Protein Levels, and Enzyme Activity in the Hippocampus

**Figure 5:**
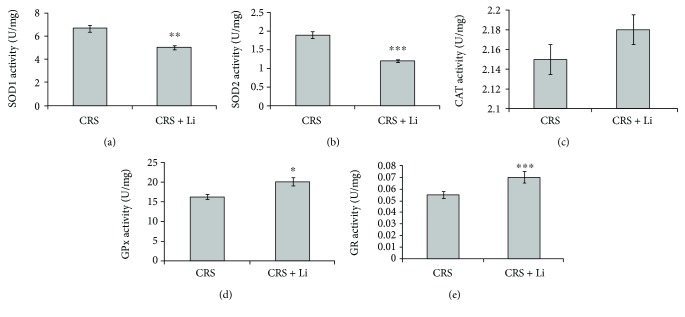
Effects of lithium on enzyme activity from CuZn superoxide dismutase (SOD1) (a), Mn superoxide dismutase (SOD2) (b), catalase (CAT) (c), glutathione peroxidase (GPx) (d), and glutathione reductase (GR) (e) in the hippocampus of animals exposed to CRS. The values are means ± S.E.M. of 10 rats. Statistical significance: ^∗^*p* < 0.05, ^∗∗^*p* < 0.01, and ^∗∗∗^*p* < 0.001 animals exposed to CRS+Li *vs*. CRS animals (*t*-test). The final result for enzyme activity was expressed as units per milligram of protein (U/mg).

**Figure 6:**
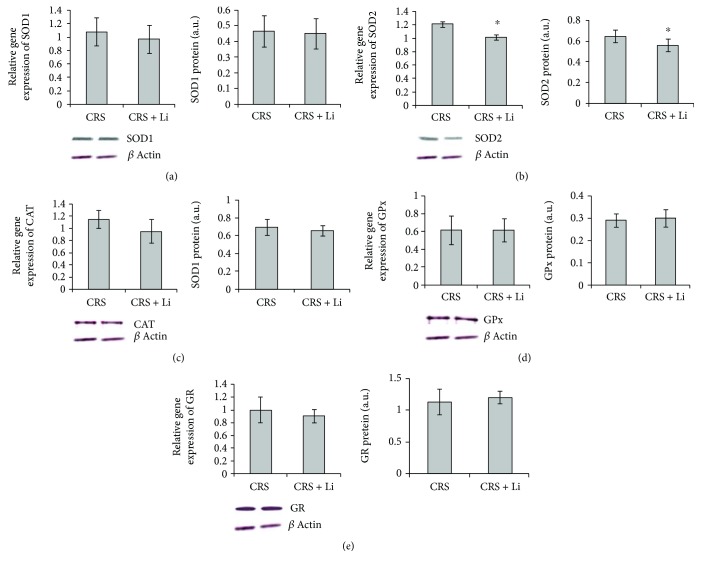
Effects of lithium on mRNA and protein levels from CuZn superoxide dismutase (SOD1) (a), Mn superoxide dismutase (SOD2) (b), catalase (CAT) (c), glutathione peroxidase (GPx) (d) and glutathione reductase (GR) (e) in the hippocampus of animals exposed to CRS. The values are means ± S.E.M. of 10 rats. Statistical significance: ^∗^*p* < 0.05 animals exposed to CRS + Li *vs*. CRS animals (t-test). The final result was expressed as fold change relative to the calibrator and normalized to cyclophilin A and protein levels was expressed in arbitrary units normalized in relation to *β*-actin.

Lithium treatment in animals exposed to CRS significantly decreased the enzyme activity of SOD1 by 25% (*p* < 0.01, *t*-test, Figure 5(a)), while levels of mRNA and protein (Figure 6(a)) remained unchanged compared with CRS animals.

### 3.4. Changes of SOD2 mRNA Levels, Protein Levels, and Enzyme Activity in the Hippocampus

The animals exposed to CRS treated with lithium showed decreased levels of SOD2 mRNA by 16% (*p* < 0.05, *t*-test, Figure 6(b)) and protein by 14% (*p* < 0.05, *t*-test, Figure 6(b)) and the enzyme activity by 37% (*p* < 0.001, *t*-test, Figure 5(b)) compared with CRS animals.

### 3.5. Changes of CAT mRNA Levels, Protein Levels, and Enzyme Activity in the Hippocampus

Lithium treatment did not change significantly gene expression and enzyme activity of CAT in animals exposed to CRS (Figures 5(c) and 6(c)).

The animals exposed to CRS treated with lithium showed a decreased ratio of SOD1/CAT and SOD2/CAT compared with CRS animals.

### 3.6. Changes of GPx mRNA Levels, Protein Levels, and Enzyme Activity in the Hippocampus

We found that lithium treatment in animals exposed to CRS significantly increased the enzyme activity of GPx by 23% (*p* < 0.05, *t*-test, Figure 5(d)), while levels of GPx mRNA and protein (Figure 6(d)) remained unchanged compared with CRS animals.

### 3.7. Changes of GR mRNA Levels, Protein Levels, and Enzyme Activity in the Hippocampus

Lithium treatment did not change the gene expression of GR enzymes (Figure 6(e)), but it significantly increased the enzyme activity of GR by 27% (*p* < 0.001, *t*-test, Figure 5(e)) in animals exposed to CRS.

### 3.8. Changes in Animal Behavior

**Figure 7:**
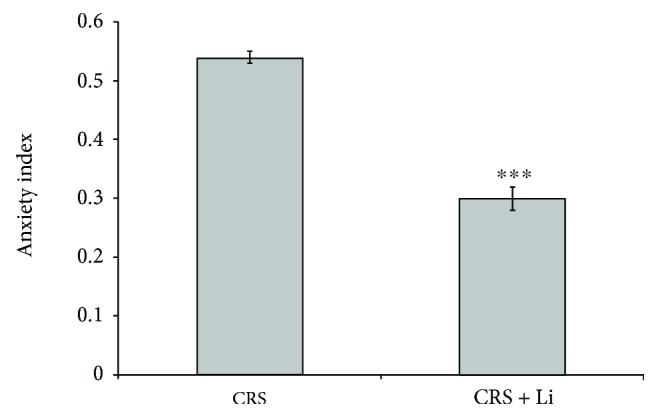
Effects of lithium on the anxiety index (AI) in animals exposed to CRS. The values are means ± S.E.M. of 10 rats. Statistical significance: ^∗∗∗^*p* < 0.001 animals exposed to CRS + Li *vs*. CRS animals (t-test).

The animals exposed to CRS treated with lithium showed significant increase of time spent in open arms compared to CRS rats. Based on these results, we calculated anxiety index (AI). Lithium treatment in animals exposed to CRS significantly decreased AI by 45% (*p* < 0.001, *t*-test, Figure 7), compared with CRS animals.

## 4. Discussion

The results of this study show that mood stabilizer lithium modulates hippocampal levels of BDNF, turnover of DA, and antioxidant defense system and stabilizes behavior in chronically stressed rats. We observed that CRS increased hippocampal BDNF protein, the key neurotrophic factor involved in the regulation of the release of neurotransmitters. This adaptive response is probably necessary to maintain the hippocampal BDNF capacity in conditions provoked by CRS because the hippocampus is the region that plays a crucial role in learning and memory and it is an area also particularly susceptible to chronic stress [8, 9]. However, lithium treatment decreased high protein levels of BDNF in chronically stressed rats to the level of unstressed animals. Our results show that it is possible that lithium has an effect on normalizing neuroplasticity in chronically stressed rats. In addition, in our earlier studies, we found that CRS induced a significant decrease of hippocampal DA concentration [6]. Literature data have confirmed that the decreased concentration of DA was observed in many psychiatric and neurodegenerative disorders, for example, depressive illness and Parkinson's disease [19, 38]. It is known that an increase of monoamine neurotransmitter levels is an important therapeutic strategy for several neuropsychiatric disorders [39]. In the present study, we found that lithium treatment increased both protein levels of hippocampal TH and concentration of DA in chronically stressed rats to the levels found in unstressed animals [6, 7], which indicates that lithium enabled *de novo* synthesis of hippocampal DA in chronically stressed rats. Lithium may have induced the gene expression of hippocampal TH in stress condition through the activator protein-1 (AP-1) transcription factor pathway [40]. The dynamics of DA transmission is regulated by reuptake through DAT. Dopamine transporter (DAT) is localized in the plasma membrane of axon terminals, and it reuptakes DA from the synapse [41] and controls the levels of DA in the extracellular space [42–44]. In this study, we found that CRS significantly increased protein levels of DAT. The higher protein levels of DAT suggest that DAT can be upregulated in response to a heightened demand for uptake of DA in conditions provoked by CRS. Stress-induced changes in the degradation of nonvesicular DA may play a role in the decrease of DA transmission. This is in line with the monoamine hypothesis of depression which states that depressive disorder is caused by insufficient signaling by monoamines [45]. An important result of this study is that lithium treatment in animals exposed to CRS decreased high protein levels of hippocampal DAT to the level of unstressed animals. It is possible that lithium has an effect on normalizing DA transmission in chronically stressed rats. In addition, monitoring of DA degradation is important for understanding dopaminergic turnover. The metabolism of monoamines by MAO is the major source of hydrogen peroxide in the brain [46]. In our previous studies, we found that CRS induced significant increase of enzyme activity of MAO B, as well as levels of COMT protein in the hippocampus [7]. These findings suggest the possibility of increased degradation of monoamine in the hippocampus in chronically stressed rats [7]. Mallajosyula et al. [47] have shown that increased MAO B activity in the astrocytes causes Parkinsonian. It is known that the inhibition of MAO activity can prolong the time during which neurotransmitters are available in the synaptic cleft [48]. Therefore, the inhibition of MAO and/or increase of monoamine neurotransmitter levels are important therapeutic strategies for several neuropsychiatric disorders [39]. The literature data confirm that lithium is a very weak inhibitor of MAO. In the present study, we found that lithium treatment in animals exposed to CRS decreased hippocampal MAO B activity to the level of unstressed animals, while levels of COMT protein remained unchanged. Decreased enzyme activity of MAO B suggests the possibility of decreased degradation of DA, which is confirmed by significantly increased concentration of DA in the hippocampus of chronically stressed rats. Our results are in accordance with the reports of Cesura and Pletscher [39] and Knoll [49] who found that the increase in DA levels was caused by MAO B inhibitors. Reduced enzymatic activity of MAO could contribute to slowing, halting, and possible reversing of neurodegeneration in dopaminergic neurons which was initiated by oxidative stress [50]. In pathological conditions, lithium treatment significantly reduces the levels of plasma lipid peroxides and improves antioxidant status [51, 52]. Decreased hippocampal MDA concentration in chronically stressed rats treated with lithium, found in this study, confirms that lithium is involved in the reduction of oxidative stress in chronic stress conditions. Based on our results, it could be speculated that decreased DA degradation via MAO may be the way by which lithium reduces oxidative stress in stress conditions. These findings suggest that further investigation of metabolites incurred from oxidative deamination of DA is needed to highlight the exact reason for reduced oxidative stress.

Modulated activities of antioxidative enzymes SOD, CAT, and GPx could be markers of oxidative stress. For example, a level of SOD is decreased when stress conditions are reduced [53]. The literature data confirm that treatment with lithium increased mRNA expression of nuclear factor (erythroid-derived 2)-like 2 (Nrf2), a signaling molecule which plays an intermediary role in defending against oxidative stress, by orchestrating the gene transcriptions of antioxidant enzymes [54–57]. In addition, nuclear factor *κ*B (NF-*κ*B) is regulated by redox sensitive factors. The absence of Nrf2 is associated with increased oxidative stress, leading to the amplification of cytokine production, as NF-*κ*B is more readily activated in oxidative environments [58]. The imbalance between Nrf2 and NF-*κ*B pathways is associated with a significant number of diseases including neurodegeneration [59]. In the present study, we observed that lithium treatment did not change the gene expression of SOD1, CAT, GPx, and GR but it decreased the gene expression of SOD2 in the hippocampus of chronically stressed rats. It is possible that the treatment with lithium is involved in maintaining a constant level of the gene expression of SOD1, CAT, GPx, and GR in chronically stressed rats for regulating the redox balance and responses to chronic stress. Furthermore, we recorded that the animals exposed to CRS treated with lithium showed a decrease of the enzyme activities of SOD1 and SOD2, while CAT activity remained unchanged. Our results are consistent with the reports of Khairova et al. [53] who also found a decrease in SOD levels, as well as unchanged CAT levels after lithium treatment. It is known that elevated SOD/CAT ratio suggests an increase in oxidative stress levels, mostly associated with the elevation in cell hydrogen peroxide concentration [60]. We recorded a reduction in SOD1/CAT ratio and SOD2/CAT ratio in animals exposed to CRS after lithium treatment, compared with CRS animals. This finding confirms that the reduction in SOD/CAT ratio may indicate lower oxidative stress, which is reflected mainly in a decrease in the concentration of cell hydrogen peroxide [60].

It is known that treatment with lithium inhibits reactive oxygen metabolite H_2_O_2_-induced cell death in primary cultured rat cerebral cortical cells, suggesting that lithium produces a protective effect against oxidative stress-induced cell death [61]. Glutathione (GSH) plays an important role in the cellular defense against ROS-induced oxidative damage in the brain [61]. Cui et al. [61] found that chronic treatment with lithium increased levels of GSH. The increased activity of GPx found in our study indicates the increased reduction of lipid hydroperoxides to their corresponding alcohols and free hydrogen peroxide to water in chronically stressed rats treated with lithium. It is possible that the increased activity of GPx compensated the decreased antioxidant capacity of CAT [62] in chronically stressed animals treated with lithium. Oxidized GSH can be reduced back by GR. In the present study, we found that treatment with lithium in animals exposed to CRS significantly increased the enzymatic activities of GR. Significantly increased enzymatic activities of hippocampal GR indicate increased reduction of GSH. It is known that the ratio of reduced GSH to oxidized GSH within cells is often used as a measure of cellular oxidative stress. Increased GPx and GR activities may also be the way by which lithium is involved in the reduction of oxidative damage in chronically stressed rats treated with this drug.

In addition, chronic treatment with mood stabilizing drug lithium in the animals exposed to CRS significantly increased time spent in open arms. This finding confirms that lithium stabilizes behavior in animals with anxiety-like behavior.

In summary, the modulation of hippocampal antioxidant status and reduced oxidative stress by lithium stabilized behavior in animals with high anxiety index. In addition, reduced oxidative stress was followed by the changes of both dopaminergic turnover and levels of BDNF protein in chronically stressed rats treated with lithium. These findings may be very important in the research on the effects of lithium on the modulation of antioxidant defense system in stress-induced diseases.

## Data Availability

The data used to support the findings of this study have been deposited in the University Library “Svetozar Marković” (Belgrade, Serbia) repository UDC number [615.214.23: 546.34]: 616.895 (043.3).
